# Effects of autonomic nervous system disorders on male infertility

**DOI:** 10.3389/fneur.2023.1277795

**Published:** 2023-10-23

**Authors:** Qixiang Qiu, Jincong Chen, Nengquan Xu, Xiaolong Zhou, Chenlian Ye, Min Liu, Zhaoxia Liu

**Affiliations:** ^1^Center for Molecular Pathology, The First Affiliated Hospital, Gannan Medical University, Ganzhou, Jiangxi, China; ^2^Department of Basic Medicine, Gannan Medical University, Ganzhou, Jiangxi, China; ^3^Center for Reproductive Medicine, The First Affiliated Hospital of Gannan Medical University, Ganzhou, Jiangxi, China; ^4^Department of Gynaecology and Obstetrics, The First Affiliated Hospital of Gannan Medical University, Ganzhou, Jiangxi, China

**Keywords:** autonomic nervous system, male infertility, pelvic floor nerve, reproductive function, sexual function

## Abstract

The male reproductive functions are largely regulated by the autonomic nervous system. Male sexual behavior and fertility primarily depend on the normal function of the higher neural centers related to the autonomic nervous system, the hypothalamic–pituitary–gonadal axis, the autonomic nervous components within the spinal cord and spinal nerves, and certain somatic nerves in the pelvic floor. In this review article, we will summarize the role of the autonomic nervous system in regulating male reproductive capabilities and fertility, its impact on male infertility under abnormal conditions, including the role of drug-induced autonomic nervous dysfunctions on male infertility. The main purpose of this article was to provide an overview of the effects of autonomic nervous dysfunction on male reproductive function and shed light on the potential therapeutic target for male infertility.

## 1. Introduction

Nearly one-seventh of couples suffer from infertility, with up to half of these cases attributed to male infertility (1). Male infertility refers to the condition in which a man, under normal reproductive conditions, is unable to facilitate pregnancy in a female partner. Normally, male fertility primarily depends on adequate sperm production and delivery to the female partner's uterus and fallopian tubes to fertilize a mature egg at ovulation. Factors leading to male infertility can be diverse and involve testicular dysfunction, endocrinopathies, lifestyle factors (such as tobacco and obesity), congenital anatomical factors, and aging (2). Any disturbances affecting sperm production, ejaculation, or transport can lead to male infertility (3, 4). Among the causes, dysregulation of the autonomic nervous system and related diseases plays a significant role in male infertility.

Normal sexual and reproductive functions are modulated by the nervous system. Neural structures involved in sexual activity span the entire nervous system, including both central and peripheral components. Among them, the peripheral nerves are divided into somatic nerves and visceral nerves. The motor nerves in the visceral nerves are called autonomous nervous systems. Its primary function is to control and regulate the body's metabolism. It is primarily involved in visceral functions such as cardiovascular, respiratory, digestive, urinary, reproductive, and endocrine functions. It is also closely related to psychological and emotional activities in humans. Dysregulation of the autonomic nervous system can potentially affect sperm generation and sperm quality through abnormalities in cardiovascular and endocrine activities. It may also impact sexual function including libido, erection, and ejaculation due to disturbances in cardiovascular and neuro-psychological activities. In the central nervous system, the higher centers related to the autonomic nervous system that are involved in male reproductive function include the anteromedial prefrontal cortex, the limbic system, and the brainstem among others. Located in the spinal cord, the lowest centers of the autonomic nervous system involved in male reproductive function include the intermediolateral nucleus of the thoracolumbar spinal cord and the sacral parasympathetic nucleus. Peripheral components of the autonomic nervous system, such as the sympathetic trunk, hypogastric plexus, and pelvic plexus, are also involved in male reproductive function. Typically, autonomic nervous system and related disorders can result in infertility through reduced sexual arousal and desire, erectile dysfunction, ejaculatory dysfunction, and semen abnormalities. The potential mechanisms of autonomic nervous system affecting on male reproductive function can be attributed to the following three main areas: First, disorders of the higher neural centers lead to emotional cognition issues, such as sexual inhibition and decreased libido. Second, disturbances in the hypothalamus–pituitary–gonadal axis (HPG axis) can affect sperm production and development, leading to male infertility. Third, impairments of the autonomic nervous components in the spinal cord and spinal nerves can result in erection and ejaculatory dysfunction in male patients (3). In this review article, we aimed to summarize the role of the autonomic nervous system in regulating male reproductive capability and the impact of autonomic nervous system disorders and related conditions on male fertility.

## 2. Autonomic nervous system disorders and male infertility

The autonomic nervous system regulates essential vital activities in conjunction with the endocrine system and various central and peripheral neural systems. Its primary function is to maintain internal homeostasis, modulating unconsciously controlled functions such as blood pressure, heart rate, respiration, body temperature, glandular secretion, digestion, and reproduction.

### 2.1. Autonomic-related higher centers involving regulating sexual desire and arousal

**Figure 1:**
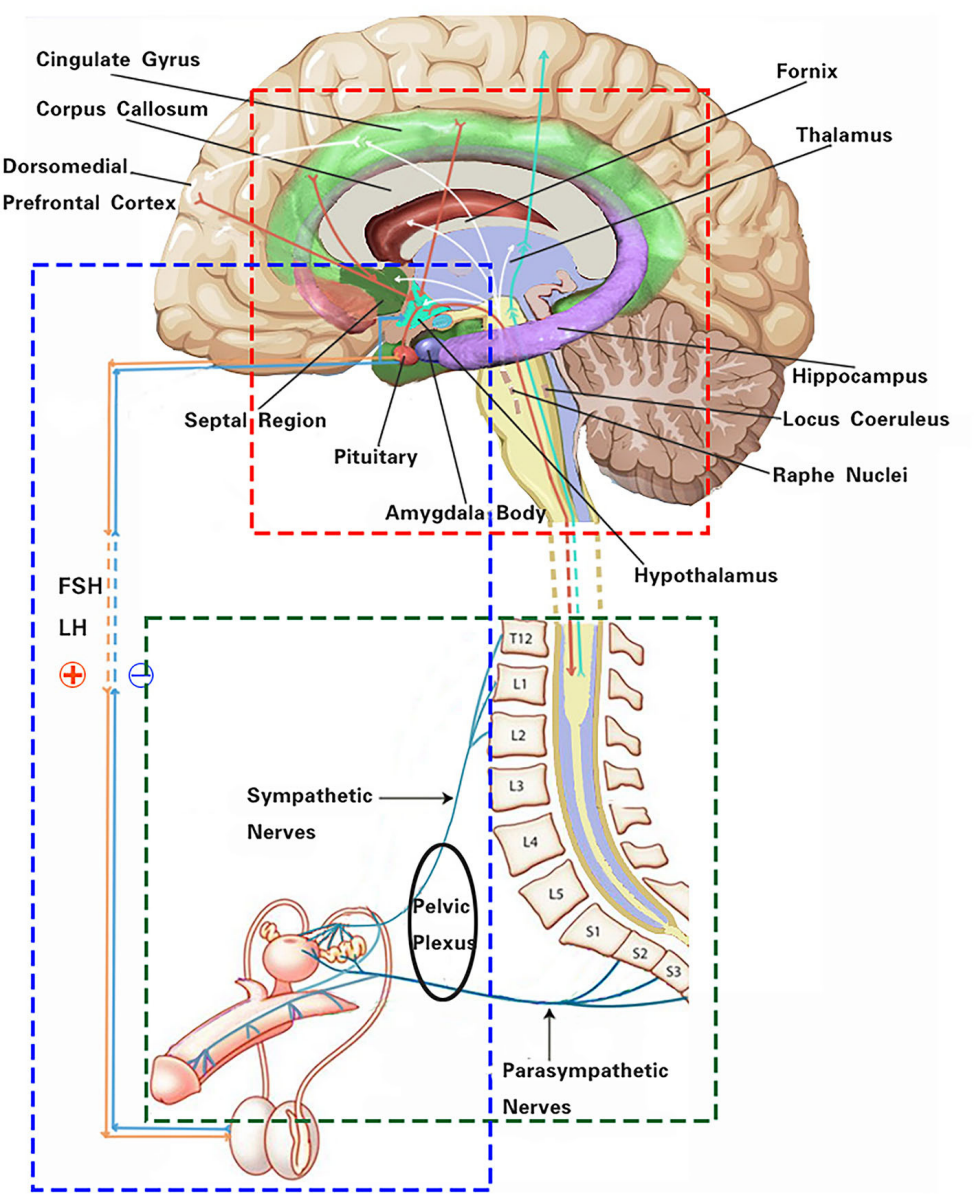
Schematic of the autonomic nervous system effect on male reproductive function. The red box indicates the main autonomic-related higher neural centers and some functional linkages involved in male reproductive function. The green box represents neural pathways of the autonomic nervous system regulating erectile and ejaculatory functions. The blue box highlights neuroendocrine pathways of the hypothalamus–pituitary–gonadal axis regulating male reproductive activity.

Several autonomic-related higher neural centers are involved in processing sexual stimuli and regulating appropriate responses to sexual desire (Figure 1). The dorsomedial prefrontal cortex plays a regulatory role in the decision-making processes of autonomic responses and emotional associations, and it participates in the inhibition of sexual arousal. Its mechanism primarily involves diverting attention and behavior to non-sexual stimuli or situations. At the same time, it mediates the effects of opiate-like drugs related to sexual reward states and endogenous cannabinoids that induce sedation (5, 6). The insular cortex, to some extent, recognizes the engorgement of the erectile organs and modulates sexual impulses, promoting attention and working memory (7, 8). The cingulate gyrus, associated with several key features of sexual behavior, serves as a key relay structure between the subcortical limbic system and the liaison cortex. It conveys integrated information to the brainstem and spinal cord to regulate autonomic nervous responses, processing sexual stimuli in conflictual contexts (9). Sexual drive and the experience of pleasure are primary contributors to initiating and sustaining sexual activity. A patient with symptomatic epilepsy due to an insular lesion reported genital arousal impairment, which was confirmed by fMRI (10). The experience of these sensations is dependent on dopaminergic neurons, mainly located in the midbrain, within the reward system (substantia nigra compact part—SNC, ventral tegmental area—VTA), and the interacting opioid–endogenous cannabinoid system. Additionally, sexual behavior requires implicit sensory stimuli, which are assessed for their sexual salience in comparison to past experiences, subsequently inducing motivational states and emotional responses. The anatomical basis for these processes encompasses limbic forebrain structures, including the cingulate gyrus, thalamus, amygdala, hippocampus, and septal nuclei (9). The thalamus is perceived as a pivotal structure in the reward system, acting not only as a relay center from the spinal cord to the cortex but also as an integrative hub, showing increased activity during phases of sexual desire, arousal, and orgasm. The amygdala assigns emotional significance to impending sexual stimuli and modulates sexual drive. Animal studies have clearly indicated the amygdala as a key structure in regulating sexual behavior. In humans, evidence of the amygdala's role in sexual function is derived from lesion studies, which indicate that stimulating the amygdala elicits sensations akin to orgasm (11).

### 2.2. Hypothalamic–pituitary–gonadal axis related disorders and male infertility

The hypothalamus, as one of the higher centers of the autonomic nervous system, exerts its regulatory functions on male reproductive function partially through neural innervation pathways. More crucially, it operates through neuroendocrine mechanisms. Male reproductive function results from the fine-tuning of several hormones involved in sexual function and spermatogenesis. For this reason, the hypothalamus, pituitary, and testes act in concert, and they are often described collectively as the hypothalamic–pituitary–gonadal (HPG) axis. After puberty, the hypothalamic neurons pulsatilely release gonadotropin-releasing hormone (GnRH), which stimulates cells in the anterior pituitary to produce luteinizing hormone (LH) and follicle-stimulating hormone (FSH). In men, the primary function of FSH is to induce Sertoli cells to produce paracrine growth factors and other elements that support spermatogenesis (12). Concurrently, LH stimulates Leydig cells to produce testosterone, which in turn, through the Sertoli cells, supports spermatogenesis. With age, there is a gradual impairment of the HPG axis, leading to a decline in testosterone levels. This decline, known as age-related male hypogonadism, can result in decreased sperm production, reduced sperm quality, and impaired fertility. Additionally, changes in hormonal levels can contribute to erectile dysfunction, further affecting reproductive ability. Despite these age-related changes in the HPG axis, endocrine functions are generally sufficient to maintain fertility in elderly men. However, understanding the impact of age-related changes on the HPG axis can help identify potential interventions to maintain and improve male reproductive health (13).

While the hypothalamus and pituitary gland are instrumental in regulating sex hormones, they also undergo feedback modulation by sex steroids, such as estradiol, testosterone, and dihydrotestosterone (14) (Figure 1). The secretion of GnRH is also regulated by negative feedback mechanisms involving inhibin B (3, 15). In obese male patients, the production of inhibin B is suppressed, disrupting the feedback loop of the HPG axis. Leptin induces the infiltration of macrophages into tissues, further triggering an inflammatory response. This can potentially lead to hypothalamic inflammation, affecting the release of hormones from the hypothalamus and causing dysregulation of the HPG axis (16). In obese patients, elevated estrogen levels, resulting from the aromatization of testosterone in the peripheral adipose tissue, exert a negative feedback on the HPG axis. This results in reduced gonadal hormones, leading to hypogonadism and hyperestrogenic hypogonadism (17).

Evidence suggests that neuroendocrine signaling pathways connect the male reproductive system with central nervous system regions, such as the limbic system and the hypothalamus (Figure 1), to respond to both acute and chronic psychological stress. There appears to be a close relationship between the testes and the stress system. Chronic stress can disrupt the normal functioning of the HPG axis, leading to alterations in the secretion of hormones crucial for reproductive processes. Prolonged stress stimuli also result in an increased secretion of glucocorticoids, which directly inhibit the secretion of growth hormone. This inhibitory effect may influence the production of IGF1 in Sertoli cells, thereby impacting early spermatogenic cells and secondary spermatocytes during spermatogenesis. Chronic stress stimuli also lead to a significant increase in NO concentration in seminal plasma, subsequently resulting in decreased sperm density, forward motility rate, and seminal plasma arginine esterase activity (18).

Tumors or focal lesions of the hypothalamus and pituitary can have adverse effects on fertility. Clinically, the most common scenarios involve pituitary adenomas or tumors. Both functional and non-functional macroadenomas of the pituitary gland result in local tumor expansion. By compressing the anterior pituitary tissue or the pituitary stalk, they can compromise gonadal function and fertility, leading to hypopituitarism and hyperprolactinemia. Oversecretion of PRL, GH, ACTH, and/or TSH by the tumor can also impair fertility. Furthermore, various therapies aimed at controlling tumor growth and inappropriate hormone secretion can interfere with reproduction. While radiation therapy has detrimental effects, surgical and chemotherapeutic interventions can either restore or further deteriorate gonadal function and fertility (19).

Several studies indicate that diabetic men might face a detrimental impact on male sexual function due to autonomic neuropathy and concomitant vascular diseases, leading to a high incidence of erectile dysfunction. Moreover, neural deficits may result in delayed ejaculation, retrograde ejaculation, and direct ejaculation with pronounced functional impairments (20). Both erectile and ejaculatory capabilities decline progressively with the advancement of autonomic neuropathy. Diabetic men may also experience a reduction in semen quality due to impairments in the HPG axis (21, 22).

Men with epilepsy may be at an increased risk of infertility due to a reduction in sperm count and decreased sperm vitality (23). The mechanism behind this reproductive dysfunction is believed to be associated with disruptions in endocrine balance due to electrophysiological effects, mainly affecting the function of the hypothalamus and pituitary. Researchers have observed impairments in gonadotropin-releasing hormone function in patients with epilepsy, elevations in LH, FSH, and prolactin levels, and a decrease in free testosterone levels (24). Apart from the direct effects of the disease, antiepileptic drugs might also adversely affect reproductive function by altering testosterone homeostasis and having a direct impact on sperm cells (25).

### 2.3. Implications of autonomic component injury in the spinal cord and spinal nerves on male reproductive function

In male reproductive function, the spinal cord plays a main role in penile erection, ejaculation, secretion from the accessory glands, and rhythmic contraction of the perineal muscles. It should be noted that the parasympathetic system within the autonomic nervous system is related to erection, while the sympathetic system is associated with ejaculation and sexual climax.

The sympathetic lowest centers are located in the intermediolateral nucleus of the spinal thoracolumbar segments T10 to L2. The parasympathetic lowest center is located in the sacral parasympathetic nucleus of spinal segments S2 to S4, while the somatic nerves originate from Onuf's nucleus in the lumbosacral region of the spinal cord (26). Prior to ejaculation, under the control of the autonomic nervous system, sperms are transported from the tail of the epididymis to the ampulla of the vas deferens through the contraction of smooth muscle tissues. When provided with sufficient stimulation, sympathetic efferent fibers from spinal segments T10 to L2 are activated. These fibers innervate the smooth muscle cells in the epididymis, vas deferens, prostate, and seminal vesicles, leading to their contraction. Through the coordinated peristaltic contraction of these structures, sperms and the seminal fluid are transported to the posterior urethra, a process referred to as ejaculation. This is followed by a sympathetic-mediated contraction of the bladder neck, while the external urethral sphincter relaxes through the coordinated action of parasympathetic fibers from spinal segments S2-4. Penile innervation arises from the autonomic nervous system (sympathetic and parasympathetic) as well as from somatic nerves that provide sensory and motor innervation (Figure 1). It is noteworthy that erection and ejaculation rely on two entirely separate neural processes; this means that an erection is not a prerequisite for ejaculation and fertility (3).

A research study has found a significant positive correlation between over activity of the autonomic nervous system and prostate size (27). The sympathetic nervous system can promote the early onset of prostate cancer, while the parasympathetic nerve fibers promote the metastasis of the cancer (28). Prostate cancer and benign prostatic hyperplasia can lead to urinary obstruction or retention, which may affect the hindered expulsion of sperm, thus affecting the quality and quantity of sperm. In addition, benign prostatic hyperplasia may cause a decrease in prostate fluid secretion or changes in its quality, which can further impact the quality of semen. These factors can potentially have a negative impact on male fertility, leading to infertility issues (29).

In men with spinal cord injuries, the nerves responsible for erection and ejaculation may be partially or completely damaged. Consequently, sexual dysfunction and infertility are exceedingly common among male spinal cord injury patients, with approximately 90% of men with spinal cord injuries unable to ejaculate through masturbation or regular sexual intercourse (30). While many men with spinal cord injuries can achieve reflexive erections and engage in intercourse, they cannot procreate without medical assistance. Beyond ejaculatory dysfunction, men with spinal cord injuries also experience a decline in semen quality, with a reduced sperm count and vitality (31). Furthermore, the semen of men with spinal cord injuries shows greater DNA damage compared to that of men in their reproductive age (32). Researchers have explored various reasons for the compromised semen quality in men with spinal cord injuries. These include alterations in prostaglandins, inflammatory cytokines, inflammatory amylosomes, changes in seminal plasma protein content, dysfunction of the accessory glands, bladder dyscoordination, over-activation of the immune system, and prostatic exhaustion (33–35). Abnormalities in semen quality might also be influenced by indirect factors and lifestyle changes, such as the severity of the spinal cord injury, testicular overheating, bladder management, or specific ejaculation techniques (33).

Multiple sclerosis (MS) is the most common chronic inflammatory demyelinating disease of the central nervous system. Due to the high dependency on the spinal reflex centers, issues with erection and ejaculation are prevalent among male MS patients. Approximately 70% of men experience erectile dysfunction, and approximately 50% of men experience retrograde ejaculation, delayed ejaculation, or anejaculation (36, 37). A study demonstrated that MS patients, compared to healthy men, exhibit reduced semen quality, decreased sperm count, diminished sperm vitality, and alterations in sperm morphology (38). Notably, as the disease progresses, the decline in semen quality becomes particularly significant in men. Potential explanations for these issues include dysregulation of the HPG axis. Other explanations encompass a reduction in sex hormone levels due to neuronal damage or chronic inflammation as well as the direct impact of autoimmune pathology on semen quality. It is even worse that the side effects of anti-anxiety and antidepressant medications might further diminish erectile and ejaculatory functions (38, 39).

Neural tube defects (spina bifida) are relatively common congenital malformations of the spinal cord. Affecting the closure of the neural tube, these defects often involve the lumbar vertebrae and can lead to sexual dysfunctions, including erectile and ejaculatory disturbances (40). In fact, patients with congenital ejaculatory dysfunctions are sometimes diagnosed with occult congenital malformations of the lumbar spinal cord. Unfortunately, surgical interventions aimed at addressing these conditions can sometimes result in further neural damage, leading to additional sexual dysfunctions.

Additionally, injuries to the lumbar sympathetic ganglia, inferior mesenteric plexus, or nerves originating from these structures can cause varying degrees of neural damage. Such injuries can result from procedures such as aortic aneurysm repairs, coronary artery bypass grafting, and any retroperitoneal lymph node dissections. It is particularly notable that both postganglionic sympathetic nerves and the inferior mesenteric plexus are excised in the “classical” retroperitoneal lymph node dissection, effectively eliminating normal ejaculation (3).

Furthermore, the majority of nerves emanating from the spinal cord that govern male reproductive functions pass through the pelvic floor to reach male genital organs. The pelvic floor muscles are innervated by both autonomic and somatic nerve fibers, including the visceral pelvic nerves and the pudendal nerve. These two types of nerve fibers can finely modulate muscles in the pelvic floor and genitalia, including those responsible for erection, micturition, ejaculation, and voiding. Therefore, diseases or traumas affecting the pelvic or perineal regions can easily disrupt these nerves, leading to male sexual dysfunctions. A significant proportion of patients undergoing cystectomy and prostatectomy experience erectile dysfunctions due to nerve damage in the pelvic floor (41).

## 3. The impact of medication-induced autonomic dysfunction on male fertility

Several studies indicate that a prolonged use of certain drugs might be associated with an increased risk of male infertility. These drugs include, but are not limited to, antihypertensives, antidepressants, and antipsychotics. These medications, along with certain toxic substances, might interfere with the normal regulatory functions of the autonomic nervous system, consequently affecting the production of testicular hormones, sperm quality and count, as well as erectile function, leading to male infertility.

The detrimental effects of drugs on male reproductive function can be categorized into primary and secondary impairments. Primary impairments include those as follows: (1) Testicular toxicity: Drugs with testicular toxicity act directly on the testes, resulting in abnormal sperm morphology, reduced sperm count, and decreased sperm maturity and vitality. This directly impacts the fertilizing capability of the sperm and the integrity of its genetic material, potentially causing infertility, fetal miscarriage, chromosomal anomalies, and congenital malformations. (2) Effects on the hypothalamic–pituitary–gonadal axis: These drugs reduce the secretion of pituitary gonadotropins, decreasing testosterone levels. This leads to endocrine dysfunctions related to male reproduction, causing alterations in endocrine hormones and receptor levels, subsequently affecting the normal function of the reproductive system and even having adverse effects on the entire organism. The secondary impairments encompass those as follows: (1) Ejaculatory and erectile dysfunction: Some drugs can cause retrograde ejaculation and suppress spinal reflexes, leading to ejaculatory dysfunctions. Erectile dysfunction, on the other hand, is often due to the drug interfering with the normal physiological activities of nerves and blood vessels. (2) Reduced libido: Some drugs lower libido by inhibiting central and peripheral neurological mechanisms (42).

Beyond drugs, other factors such as environmental pollution and unhealthy lifestyles (e.g., smoking, excessive alcohol consumption, poor dietary habits, and mental stress) can also cause autonomic nervous system disorders, thereby negatively impacting the male reproductive system (43). A summary of currently known drugs and environmental toxins that impair male fertility by affecting the autonomic nervous system is listed in Table 1.

**Table 1:** Drugs related male infertility by inducing the autonomic nervous system dysfunction.

**Drug classification**	**Drug name**	**Disease treated by the drug**	**Effects on the autonomic nervous system**	**Effects on male infertility**	**References**
Corticosteroids	Prednisone, prednisolone, dexamethasone	Rheumatoid arthritis, asthma	Potential inhibition of the HPG (low testosterone levels)	Erectile dysfunction and decreased libido	(33, 34)
Analgesics	Tramadol	Various pain-causing diseases	Inhibition of the proerectile-related central dopaminergic pathway	Induce erectile disorders	(35)
Hormonal treatments	Triptorelin, leuprorelin, goserelin, buserelin	Prostate cancer, breast cancer	Desensitization of pituitary receptors	Erectile dysfunction and loss of libido blockage	(36)
Antidepressant therapy	Fluoxetine, paroxetine	Depression	Suppressing the activity of excitatory neurons	Affecting erectile function, ejaculation disorders, anorgasmia	(37)
Antipsychotic therapy	Chlorpromazine, haloperidol	Psychosis	Blockade of dopamine D2 receptors in the tuberoinfundibular pathway	Decrease the libido by inhibiting motivation and reward	(38)
Antiepileptic treatment	Valproate lamotrigine	Epilepsy	Raise the level of GABA in the central nervous system	Erectile dysfunction and indirectly affecting sexual excitement	(14, 39)
			Inhibiting the conduction function of the nervous system		
Tranquilizer	Benzodiazepines	Anxiety disorder, mental disorder, insomnia	Inhibition of the proerectile central dopaminergic pathway	Erectile disorders	(40)
Environmental toxins	Phthalates, organophosphates	Poisoning	Antiandrogenic	Sexual dysfunctions and decrease in sperm motility	(41–43)
			Reduce monoamine levels needed for adequate HPGA activity		

## 4. Conclusion

In conclusion, this article presents compelling evidence highlighting the crucial role of dysregulation of the autonomic nervous system and related diseases in male infertility. The autonomic nervous system, along with the related central and peripheral neural systems, plays a significant role in regulating sexual and reproductive functions. Dysfunctions within this system, some related diseases, and medication can lead to various sexual dysfunctions and semen abnormalities, impacting male fertility. The findings underscore the importance of considering the autonomic nervous system when assessing the causes of male infertility. This research has significant clinical implications, emphasizing the need for clinicians and researchers to evaluate autonomic nervous system function and related diseases in the context of male reproductive health. Future research should focus on exploring the complex mechanisms linking the autonomic nervous system and male fertility to better understand the impact on male reproductive function. Furthermore, exploring novel treatment methods and interventions to aid individuals facing reproductive difficulties due to autonomic nervous system dysregulation is an important direction for future investigations. By enhancing our understanding and addressing the effects of autonomic nervous system dysregulation on male reproductive function, we can provide more effective treatments and rehabilitation strategies for male patients.

Future research should focus on exploring the complex mechanisms linking the autonomic nervous system and male fertility to better understand the impact on male reproductive function. Furthermore, exploring novel treatment methods and interventions to aid individuals facing reproductive difficulties due to autonomic nervous system dysregulation is an important direction for future investigations. By enhancing our understanding and addressing the effects of autonomic nervous system dysregulation on male reproductive function, we can provide more effective treatments and rehabilitation strategies for male patients.

## Author contributions

QQ: Conceptualization, Data curation, Writing—original draft, Writing—review and editing. JC: Conceptualization, Writing—review and editing, Data curation. NX: Writing—review and editing. XZ: Writing—review and editing. CY: Writing—review and editing. ML: Writing—review and editing. ZL: Resources, Writing—review and editing, Conceptualization, Funding acquisition, Project administration, Supervision.
